# Treating Colon Cancer Cells with FK228 Reveals a Link between Histone Lysine Acetylation and Extensive Changes in the Cellular Proteome

**DOI:** 10.1038/srep18443

**Published:** 2015-12-17

**Authors:** Tian-yun Wang, Yan-long Jia, Xi Zhang, Qiu-li Sun, Yi-Chun Li, Jun-he Zhang, Chun-peng Zhao, Xiao-yin Wang, Li Wang

**Affiliations:** 1 Department of Biochemistry and Molecular Biology, Xinxiang Medical University, Xinxiang 453003, Henan, China; 2 Pharmacy Collage, Xinxiang Medical University, Xinxiang 453003, Henan, China; 3 Henan Collaborative Innovation Center of Molecular Diagnosis and Laboratory Medicine, Xinxiang 453003, China

## Abstract

The therapeutic value of FK228 as a cancer treatment option is well known, and various types of cancer have been shown to respond to this drug. However, the complete mechanism of FK228 and the affect it has on histone lysine acetylation and the colon cancer cell proteome are largely unknown. In the present study, we used stable isotope labeling by amino acids in cell culture (SILAC) and affinity enrichment followed by high-resolution liquid chromatograph-mass spectrometer (LC-MS)/MS analysis to quantitate the changes in the lysine acetylome in HCT-8 cells after FK228 treatment. A total of 1,194 lysine acetylation sites in 751 proteins were quantified, with 115 of the sites in 85 proteins being significantly upregulated and 38 of the sites in 32 proteins being significantly downregulated in response to FK228 treatment. Interestingly, 47 histone lysine acetylation sites were identified in the core histone proteins. We also found a novel lysine acetylation site on H2BK121. These significantly altered proteins are involved in multiple biological functions as well as a myriad of metabolic and enzyme-regulated pathways. Taken together, the link between FK228 function and the downstream changes in the HCT-8 cell proteome observed in response to FK228 treatment is established.

Histone acetyltransferases (HATs) and deacetylases (HDACs) function to modify the activity of histones and play crucial roles during proliferation, apoptosis, development, angiogenesis, and carcinogenesis^1^. Furthermore, various inhibitors have been discovered to counteract the removal of the acetyl groups from histones by HDAC^2^,^3^. In fact, several HDAC inhibitors have also been shown to have strong anticancer properties, and many of these inhibitors have moved forward into clinical trials as cancer treatment options^4^,^5^,^6^,^7^.

FK228 (Romidepsin, FR901228), also known as depsipeptide, is an HDAC inhibitor that is isolated from a fermentation product of violaceina^8^,^9^ and was approved by the U.S. Food and Drug Administration for the treatment of cutaneous T-cell lymphoma (CTCL) in 2009 and peripheral T-cell lymphoma (PTCL) in 2011. In a phase II trial consisting of 71 CTCL patients who had received an average of four prior therapies, the patients demonstrated not only a good overall response rate to FK228, but also a durable response with a median response length of 13.7 months^10^. Similarly, in a phase II trial involving 47 patients with PTCL who had received a median of treatments, an overall response rate of 38% was observed following FK228 treatment, with eight complete responses^11^. The therapeutic value of FK228 during the treatment of solid tumors, including lung, pancreatic, thyroid, bladder, and esophageal cancer, has also been widely studied^12^,^13^,^14^,^15^,^16^. Moreover, previous studies investigating the mechanism of this drug have indicated that FK228 can inhibit the growth of HCT-116 cells, a human colon carcinoma cell line, *in vitro* and *vivo* more effectively than fluorouracil (5-Fu), a commonly used chemotherapeutic drug^17^. FK228 was also observed to induce apoptosis in Caco-2 cells, another colon adenocarcinoma cell line^18^. Notably, although FK228 treatment appears to have anticancer properties, the full mechanisms of this drug and its impact on epigenetic regulation and the proteome are largely unknown. The only study to make note of the changes in the protein profile following FK228 treatment was limited to H322 cells, a lung cancer cell line^19^. Thus, it is essential to further assess the downstream effects of FK228 in other cell lines in order to fully understand the function of this HDAC inhibitor in various types of cancer.

In the present study, we sought to determine if FK228 treatment does in fact alter the histone lysine acetylation profile and if these changes subsequently affect the proteome of cancer cells. To this end, we used stable isotope labeling by amino acids in cell culture (SILAC) and affinity enrichment followed by high-resolution liquid chromatograph-mass spectrometer (LC-MS)/MS analysis. To our knowledge, this is the first quantitative lysine acetylome and proteome analysis performed in HCT-8 cells following FK228 treatment.

## Results and Discussion

### Cell cytotoxicity assay

**Figure 1:**
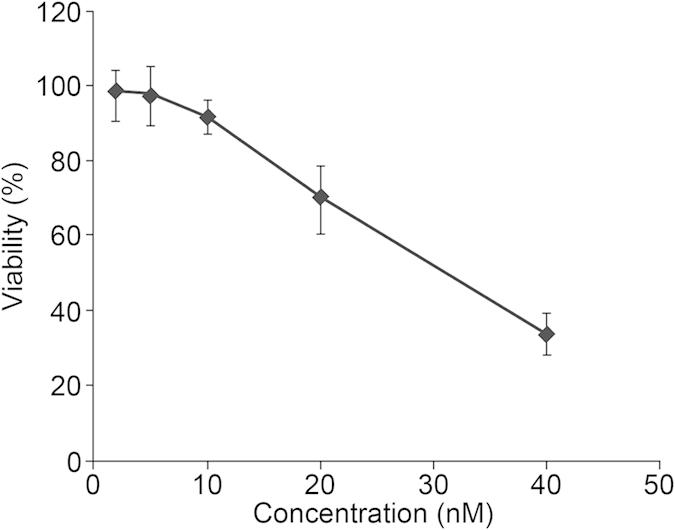
The establishment of appropriate FK228 working concentration.

To establish the appropriate FK228 treatment concentration, a cytotoxicity assay using varying concentrations of FK228 was performed. Our results demonstrate a dose-dependent response, whereby the viability of cultured HCT-8 cells was observed to decrease as the FK228 concentration increased (Fig. 1). Notably, approximately 50% of the cells were viable (IC50) at a FK228 concentration of 29.46 nM. Therefore, this dosage of FK228 was used for the 18 h treatment period for all of the subsequent experiments.

### Profile of FK228-treated proteome

Acetylation and deacetylation of histones in multiple sites has been generally associated with transcriptional activation and repression, respectively^20^,^21^. Notably, FK228 has been identified to function as an effective HDAC inhibitor and can influence histone acetylation and downstream gene expression. However, the level of histone lysine acetylation following FK228 treatment and the resulting effect on the proteome has not been investigated in HCT-8 cells. To this end, we performed quantitative proteomics to profile the differentially expressed proteins observed between untreated cells and FK228-treated cells. Altogether, this investigation identified 1,209 lysine acetylation sites in 760 protein groups, among which 1,194 sites in 751 proteins were quantified. In order to focus on the proteins with expression changes that could result in altered function, we concentrated on proteins associated with quantification ratios of greater than 2.0 and less than 0.5, using these values for the upregulated and downregulated thresholds, respectively^22^. Using these cutoffs, 115 lysine acetylation sites in 85 proteins were quantified as upregulated targets, while 38 lysine acetylation sites in 32 proteins were quantified as downregulated targets. These data indicate that FK228 treatment does in fact induce changes in the histone lysine acetylation profile and that variation at these specific sites subsequently results in an altered cellular proteome. All of the identified quantifiable proteins with expression changes greater than 2.0 fold (upregulated) or less than 0.5 fold (downregulated) are listed in Supporting Information Table S1.

### Lysine acetylation levels in the core histones are altered

HDAC inhibitors have been extensively studied in the quest to understand basic chromatin structure and transcriptional regulation, and many have been introduced as clinical treatments for cancer^23^,^24^,^25^. HDAC inhibitors are known to induce the accumulation of hyperacetylated nucleosome core histones, resulting in the transcriptional activation of various genes^23^. FK228 is a novel and effective HDAC inhibitor, and has been shown to suppress HDAC 10 times better than TSA, another well-known HDAC inhibitor, in human cancer cell lines^26^,^27^. A previous study using site-specific antiacetyllysine antibodies to study the effect of FK228 on H1299 cells transfected with mutated p53 (K319/K320/K321R mutated and K373R/K382R mutations) revealed that this inhibitor specifically induces p53 acetylation at K373/K382, but not at K320^28^. After determining that FK228 does in fact alter the lysine acetylation profile in the proteome of HCT-8 cells, we sought to determine the specific effects on the core histone proteins.

**Figure 2:**
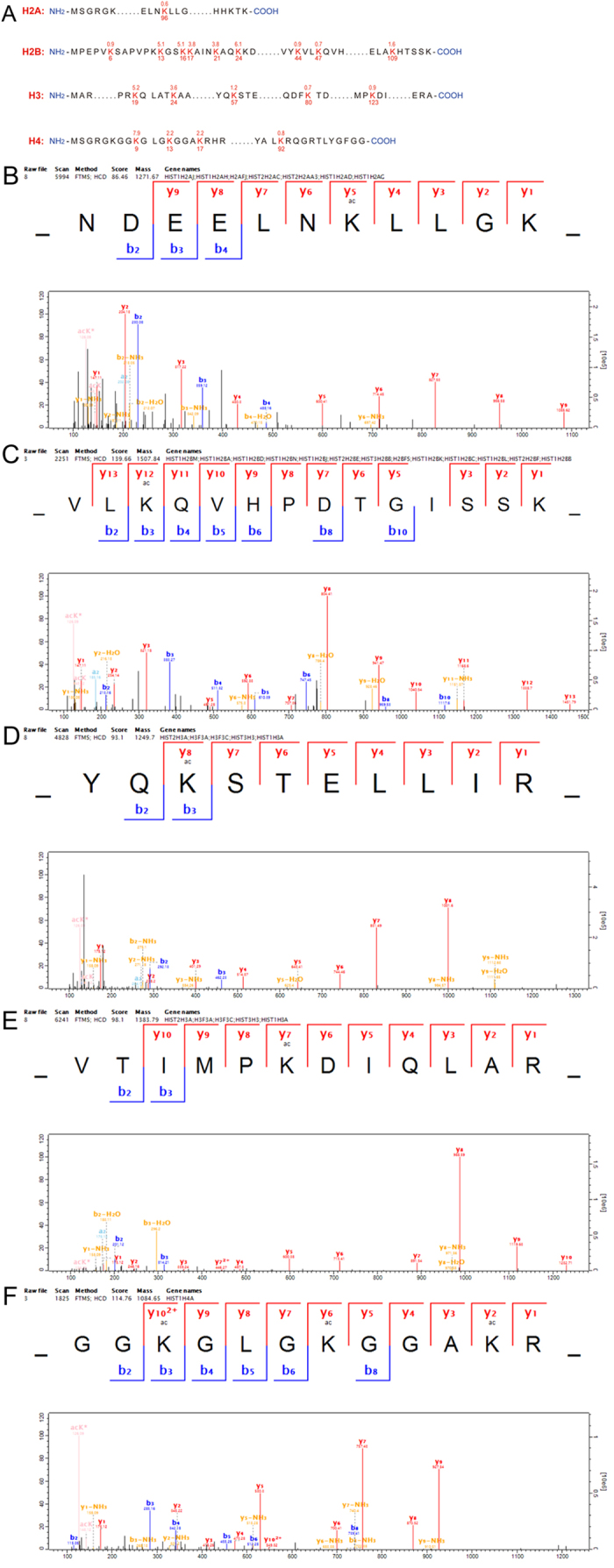
Identification and quantitation of lysine acetylation in histones in HCT-8 cells. (**A**) The illustration of identified lysine acetylation sites in core histones in HCT-8 cells in response to FK228 treatment. The identified sites in core histones were numbered and highlighted; (**B**) MS/MS spectra of a tryptic peptide ion histone H2AK96 acetylated peptide _NDEELNK(ac)LLGK_; (**C**) MS/MS spectra of a tryptic peptide ionhistone H2BK47 acetylated peptide _VLK(ac)QVHPDTGISSK_; (**D**) MS/MS spectra of a tryptic peptide ion histone H3K57 acetylated peptide _YQK(ac)STELLIR_; (**E**) MS/MS spectra of a tryptic peptide ion histone H3K123 acetylated peptide _VTIM(ox)PK(ac)DIQLAR_; (**F**) MS/MS spectra of a tryptic peptide ion histone H4K9 acetylated peptide _GGK(ac)GLGK(ac)GGAK(ac)R_.

As shown in Supporting Information Table S2, a total of 47 lysine acetylation sites in the core histones were identified, which cover almost all of the reported lysine acetylation sites in mammalian histones. The spectra for five peptides, including H2AK96ac, H2BK44ac, H3K19ac, H3K80ac, and H4K13ac, are shown in Fig. 2A–E, as they represent the spectra of the most well-known histone lysine acetylated peptides. The sequences of the identified lysine acetylated peptides in the core histones and all of their corresponding quantitative changes in acetylation following FK228 treatment are shown in Table 1. These data indicate that the H2AK5ac, H2BK5ac, H3K9ac, H4K5ac, and H4K16ac peptides were all significantly increased in response to FK228 treatment, while H2AK95ac, H2BK16ac, H3K56ac, H3K79ac, and H4K31ac showed little to no change in expression. In addition, the acetylation levels observed at some sites were markedly decreased following FK228 treatment, including that for H2BK23ac, H3K23ac, and H4K12ac. Histone modification in the N-terminus of a protein is considered a “histone code” or “epigenetic code” modification, which plays an important role in chromatin remodeling and gene transcriptional regulation^20^,^29^. In this study, most of the quantifiable lysine acetylation sites were found in the N-terminal regions of the core histone proteins, suggesting that FK228 is affecting the innate epigenetic mechanism involved in HCT-8 cell function.

**Figure 3:**
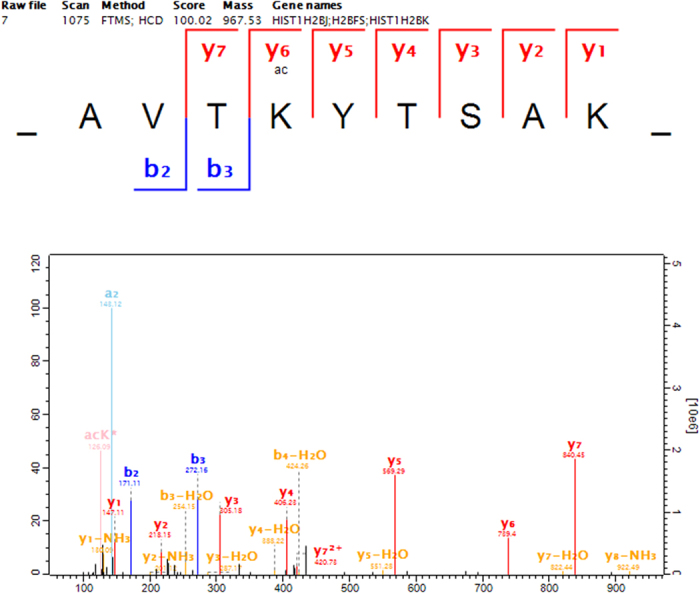
MS/MS data and annotations for the novel lysine acetylation site on H2BK121.

Notably, of the 47 histone lysine acetylation sites we identified in the HCT-8 core histones, we also detected a novel lysine acetylation site on H2BK121(Fig. 3). In fact, aside from this one novel site, the other acetylated lysine residues have been observed to occur in various species and sample types, including cells, tissues, clinic samples, etc.^30^. Further, among the quantifiable lysine acetylated sites detected, most had elevated acetylation levels. Strikingly, H2BK121 actually had a significantly decreased level of acetylation in response to FK228 treatment, indicating that FK228 may function in a non-HDAC inhibitor-related manner. Identification of this novel histone lysine acetylation site suggests that a previously unknown epigenetic mechanism may occur in response to FK228 treatment in HCT-8 cells.

**Figure 4:**
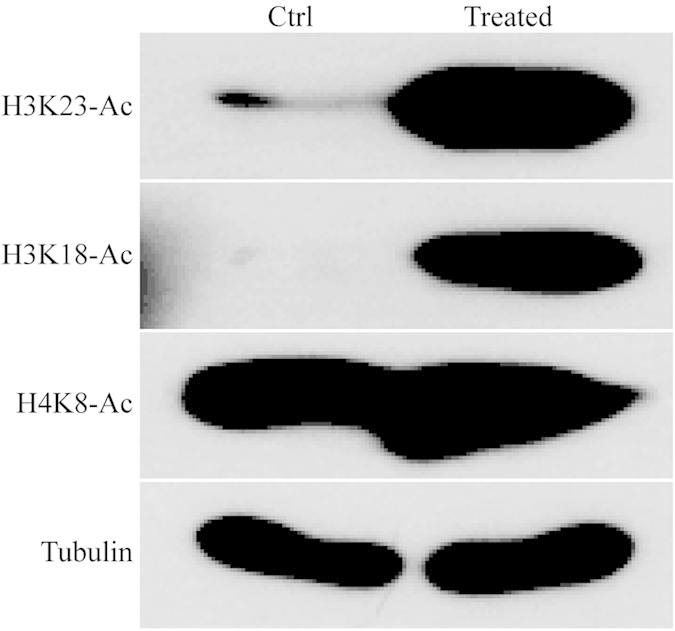
Change of histone acetylation profiles in response to FK228 treatment. HCT-8 cells were treated with or without FK228 with 1.0 μM of concentration. Cells were then harvested, and the core histones from whole cell lysates were extracted. 15 μg of extracted core histones was subject to SDS-PAGE followed by Western blotting analysis to exam core histone site-specific lysine acetylation changes in H3 and H4 (D) by using indicated histone site-specific lysine acetylation antibodies.

To further investigate the change of histone acetylation profiles in response to FK228 treatment, we next performed Western blot analysis with histone lysine acetylation sequence-specific antibodies. As shown in Fig. 4, 1.0 μM Fk228 treatment significantly increased the acetylation level in H3K23ac, H3K18ac, H4K8ac (Fig. 4), which was consistent with quantitative results summarized in Table 1.

### Characterization of differentially quantified proteins

To better understand the lysine acetylation changes in the HCT-8 proteome in response to FK228 treatment, we investigated the GO functional classification of all of the identified acetylated proteins based on their biological process and molecular function (Supporting Information Table S3). The results for the biological process analysis showed that the largest group of acetylated proteins is composed of enzymes associated with cellular process and single-organism, which accounts for 14% and 13% of the upregulated acetylated proteins and 16% and 14% of the downregulated acetylated proteins, respectively. Moreover, large portions of the acetylated proteins were also classified in the metabolic process and cellular component organization or biogenesis processes, account for 12% and 10% of the upregulated acetylated proteins and 13% and 10% of the downregulated proteins, respectively. In terms of their molecular function, the largest number of acetylated proteins was categorized in the binding proteins function (53% and 47% of the upregulated and downregulated acetylated proteins, respectively). The second largest molecular function GO group, accounting for 24% and 32% of the upregulated and downregulated acetylated proteins, was the catalytic activity group. Taken together, our GO analysis of the acetylome suggests that the acetylated proteins have a wide range of molecular functions and are involved in a myriad of biological processes in HCT-8 cells. This vast array of functions also likely influences the mechanism of FK228 treatment, allowing it to affect a number of cellular pathways.

**Figure 5:**
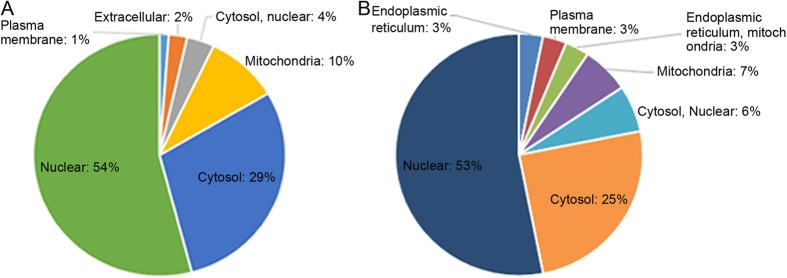
The subcellular location of up-regulated (**A**) and down-regulated (**B**) proteins (FK228-vs-FKC).

**Figure 6:**
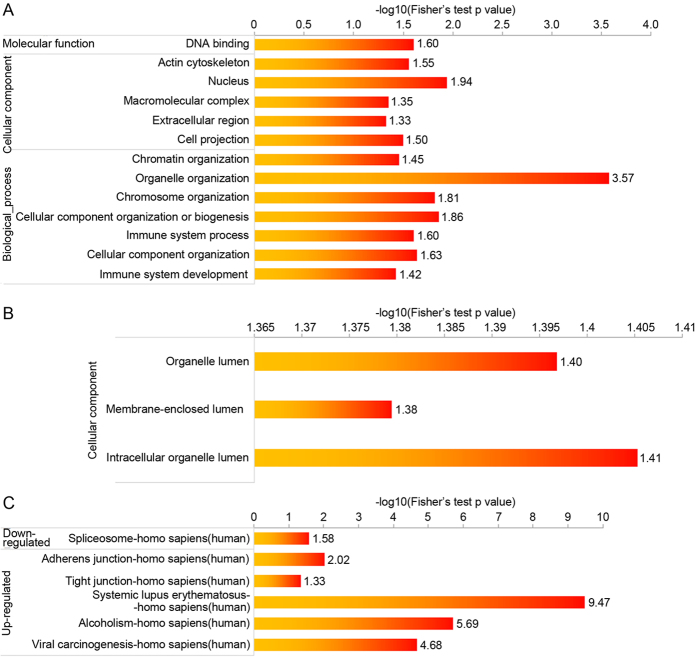
Enrichment analysis of the acetylated proteins in HCT-8 in response to FK228 treatment. GO-based enrichment analysis of up-regulated (**A**) and down-regulated (**B**) proteins (FK228-vs-FKC). (**C**) KEGG pathway-based enrichment analysis of up- and down-regulated proteins (FK228-vs-FKC).

As shown in Fig. 5, the subcellular location of the acetylated proteins was also analyzed. These results show that 54% of the upregulated acetylated proteins and 53% of the downregulated acetylated proteins are distributed in the nucleus. On the other hand, 37 proteins appeared to be located in the periplasmic space (6%), while 29% of the upregulated and 25% of the downregulated proteins are distributed in the cytosol. Furthermore, to determine which types of proteins are the preferred targets for lysine acetylation, we evaluated the enrichment levels for two GO categories: cellular component and biological process. In the upregulated proteins analyzed for their biological process, a significant level of enrichment was observed in processes related to immune system development, cellular component organization, immune system process, cellular component organization or biogenesis, chromosome organization, organelle organization, and chromatin (Fig. 6A). Using the KEGG pathway analysis, several other biological functions were also found to be enriched in the upregulated proteins, including viral carcinogenesis, adherens junction, and tight junction in the up-regulated. In terms of their cellular component, these upregulated proteins were enriched in the nucleus, DNA binding, cell projection, extracellular region, macromolecular complex, and actin cytoskeleton categories(Fig. 6A). However, in the downregulated proteins, we observed enrichment in the intracellular organelle lumen, membrane-enclosed lumen, and organelle lumen components. Moreover, proteins involved in systemic lupus erythematosus and alcoholism were also the most enriched in the downregulated acetylated proteins, as determined with the KEGG pathway analysis (Fig. 6B). Spliceosome proteins were also enriched in this group.

We also analyzed the biological activity/biological relevance of the proteins identified, and the number and proportion at the same kind of GO term were compared, the results showed that the difference showed statistically significant in enzyme regulator activity, reproduction, single-organism process, response to stimulus, cellular component organization or biogenesis (*P* < 0.05, Figure S1).

### Protein biological functions

Although our enrichment analysis allowed us to get a general understanding of the functions of the acetylated proteins regulated by FK228, we sought to further analyze the enrichment in the three GO categories, molecular function, cellular compartment, and biological process. To this end, the proteins with quantified changes in acetylation after FK228 treatment were divided into four quantiles according to their quantification ratio: Q1 (0~15%), Q2 (15~50%), Q3 (50~85%), and Q4 (85~100%).

**Figure 7:**
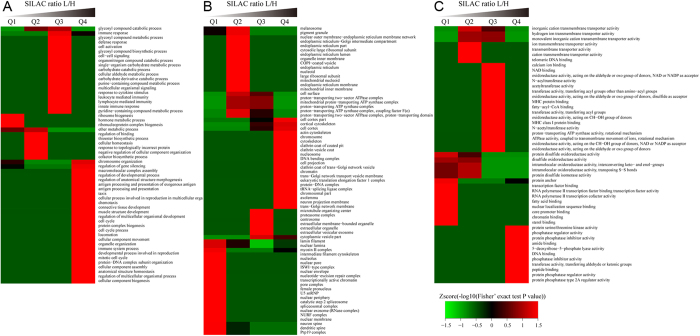
Gene ontology (GO) enrichment-based cluster analysis. Quantifiable proteins were classified by gene ontology annotation based on three categories: (**A**) molecular function; (**B**) cellular compartment, and (**C**) biological process. In each category, the quantified proteins in this study were divided into four quantiles according to the quantification ratio to generated four quantiles: Q1 (0~15%), Q2 (15~50%), Q3 (50~85%) and Q4 (85~100%). An enrichment analysis was performed separately in each quantile for diverse categories, and the overrepresented annotations were clustered through one-way hierarchical clustering for comparative analysis.

As shown in Fig. 7A, in the biological process category, proteins with high L/H ratios appear to be related to ion cell cycle DNA replication, chromatin assembly, and telomere maintenance, while the processes related to telomere maintenance, hormone metabolic process, ribonucleoprotein complex biogenesis, and ether metabolic process were rare in quantiles with high L/H ratios. These results suggest that FK228 treatment markedly affected pathways that are controlled via acetylation.

In agreement with this observation, our in depth analysis of the cellular component of these four quantiles (Fig. 7B) showed that proteins related to chromosome, cytoskeleton, nucleosome, chromatin, and DNA bending complex were significantly enriched in quantiles with high L/H ratios, while the processes related to melanosome, Prp19 complex, spliceosomal complex, mitochondrial inner membrane, and endoplasmic reticulum membrane were rare in quantiles with high L/H ratios. These data are consistent with previous data indicating that the mitochondrion is a major cellular compartment that functions in response to acetylation related events.

Moreover, the enrichment analysis of these quantiles in terms of their molecular functions (Fig. 7C) showed that proteins involved in phosphatase regulator activity, phosphatase inhibitor activity and transferase activity, and transferring aldehyde or ketonic groups were all enriched after FK228 treatment. Therefore, despite the differential expression pattern observe in the HCT-8 proteome, the enriched biological functions still appear to be tightly associated with lysine acetylation events, supporting the intrinsic role of FK228 as an HDAC inhibitor in this cell type.

### Enrichment analysis of protein domains and cellular pathways

**Figure 8:**
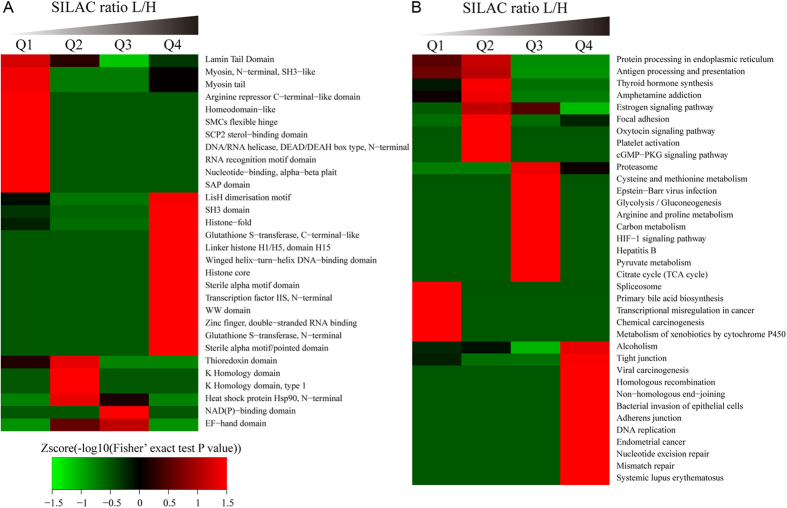
Protein domain and KEGG pathway-based clustering analysis. (**A**) The protein domain database; (**B**) The KEGG pathway database.

The specific domain structure of a protein is one of the major indicators used to identify protein function. To investigate the domain features of the proteins affected by FK228 treatment, we analyzed the Interpro domain enrichment of the four L/H ratio quantiles according to our results from the molecular function and biological process analysis. It appears that the proteins affected by FK228 treatment contain four primary domains: LisH dimerization motif, histone core, sterile alpha motif domain, and winged helix-turn-helix DNA-binding domain (Fig. 8A). Further, we also carried out a pathway clustering analysis of the FK228 responsive proteome using the pathways identified by our KEGG analysis (Fig. 8B). The results of this investigation suggest that protein expression involved in DNA replication, adherens junction, glycolysis/gluconeogenesis, and the citrate cycle were the most prominently upregulated pathways after FK228 treatment. In contrast, protein expression in the spliceosome, primary bile acid biosynthesis, and transcriptional misregulation in cancer pathways was decreased in response to FK228 treatment.

## Methods

### Cell culture

Colon cancer HCT-8 cells were purchased from the Cell Bank at the Chinese Academy of Sciences (Shanghai, China). The cells were cultured at 37°C with 5% CO_2_ in Dulbecco’s modified Eagle’s medium (DMEM) medium containing 10% fetal calf serum, 100 μg/ml penicillin, and 100 μg/ml streptomycin. The cells were subcultured every 2~3 days after digestion with 0.02% EDTA, 0.1% trypsin.

### Cell proliferation and cytotoxicity assay

When the cells reached 75% confluence, a cell proliferation and cytotoxicity assay was carried out using a Cell Counting Kit-8 according to the manufacturer’s instructions (CCK-8; Dojindo Laboratories, Kumamoto, Japan). Briefly, a 96-well plate was preincubated for 24 h in a humidified incubator, and then approximately 2,000–5,000 cells were dispensed into each well in 100 μL suspensions. Different concentrations of FK228 (Sigma, St. Louis, MO) were then prepared and added to each well, and the plate was incubated for an appropriate length of time. After this incubation period, 10 μL of CCK-8 sSolution was added to each well and incubated for an additional 1–4 h. Finally, cell viability was determined by measuring the absorbance at 450 nm using a microplate reader. Cell cytotoxicity was calculated using the measured HTC-8 cell viability following treatment with the various concentrations of FK228 for the specified treatment time. The FK228 treatment concentration resulting in 50% cell viability (IC_50_) was chosen as the fixed working concentration for all additional experiments.

### SILAC labeling

When the cultured cells achieved 80% confluence, the cells were then labeled with either “heavy isotopic lysine” (^13^C-Lysine) or “light isotopic lysine” (^12^C-Lysine) using a SILAC Protein Quantitation Kit (Pierce, Thermo). Briefly, the cells were grown in DMEM supplemented with 10% fetal bovine serum and either the “heavy” form of [U-^13^C_6_]-L-lysine or “light” [U-^12^C_6_]-L-lysine for 8 generations. After labeling was achieved, the cells were replated in 15 cell culture flasks (150cm^2^) at a concentration of approximately 5×10^8^ cells/plate in SILAC media. The “light” labeled cells were then treated with 20.36 ng/mL of FK228, while the “heavy” labeled cells were treated with the same concentration of dimethylsulphoxide (DMSO). After treatment, the cells were maintained in SILAC media for another 48 h. The cells were then harvested and washed twice with ice-cold PBS supplemented with 2 μM trichostatin A and 30 mM nicotinamide. After snap freezing in liquid nitrogen, cell pellets were stored at −80 °C until future use.

### Protein extraction

The harvested “heavy” and “light” labeled cells were lysed with 2×NETN buffer (200 mM NaCl, 2 mM EDTA, 100 mM Tris-Cl, 1.0% NP-40, pH 7.2) supplemented with 0.5% Triton X-100 on ice for 30 min. The individual supernatants were kept after centrifugation at 20,000 × g for 10 min at 4 °C. Total lysate protein concentrations were detected using the standard Bradford assay (Bio-Rad, San Diego, CA, USA)., equal amounts of crude proteins from the “heavy” and “light” supernatants were mixed and precipitated with trifluoroacetic acid (TCA) at a 15% final concentration (v/v) (soluble fraction). After washing twice with −20 °C acetone, the proteins pellets were dissolved in 100 mM NH_4_HCO_3_ (pH 8.0) for trypsin digestion. The remaining cell pellets were dissolved in 8 M urea to extract the chromatin-binding proteins. After measuring the protein concentrations in each sample, equal amounts of the isolated chromatin-binding proteins from each were mixed, followed by protein precipitation with TFA at a final concentration (v/v) of 15% (nuclear pellet fraction). After washing twice with −20 °C acetone, the protein pellets were dissolved in 100 mM NH_4_HCO_3_ for trypsin digestion.

### Trypsin digestion

Trypsin was added into protein solution at a trypsin to protein ratio of 1:50 (w/w) and digestion was performed at 37 °C for 16 h. DTT was then added at a final concentration of 5 mM, followed by incubation at 50 °C for 30 min. Subsequently, iodoacetamide (IAA) was added to alkylate proteins at a final concentration of 15mM, followed by incubation at room temperature in the dark for 30 min. This alkylation reaction was quenched using 3 mM of cysteine (final concentration) at room temperature for another 30 min. Trypsin was then added again at a trypsin to protein ratio of 1:100 (w/w) and incubated at 37 °C for 4 h to complete the digestion cycle.

### HPLC fractionation

Following trypsin digestion, the samples were then fractionated with high pH reverse-phase HPLC using an Agilent 300Extend C18 column. Briefly, peptides were first separated with a gradient of 2% to 60% acetonitrile (ACN) in 10 mM ammonium bicarbonate (pH 10) for 80 min, resulting in 80 fractions. Then, the peptides were combined into six primary fractions and dried by vacuum centrifugation.

### Affinity enrichment

To enrich the tryptic peptides, the six primary fractions were dissolved in NETN buffer were incubated with pre-washed antibody beads (PTM Biolabs, Hangzhou,China) at 4 °C overnight with gentle shaking. The beads were washed four times with NETN buffer and twice with ddH_2_O. The bound peptides were eluted from the beads with 0.1% TFA. The eluted fractions were combined and vacuum-dried. The resulting peptides were cleaned with C18 ZipTips (Millipore) according to the manufacturer’s instructions. These samples were then used for LC-MS/MS analysis.

### LC-MS/MS analysis

Peptides were dissolved in 0.1% formic acid (FA) and directly loaded onto a reversed-phase pre-column (Acclaim PepMap 100, Thermo Scientific). Peptide separation was then performed using a reversed-phase analytical column (Thermo Scientific). The separation gradient comprised of a 6% to 22% increase in solvent B (0.1% FA in 98% ACN) for 24 min, followed by an increase from 22% to 35% for 10 min, climbing to 80% in 5 min, and then holding at 80% for the last 3 min. Subsequently, the resulting peptides were analyzed with tandem mass spectrometry (MS/MS) with a Q ExactiveTM hybrid quadrupole-Orbitrap mass spectrometer (ThermoFisher Scientific) coupled online with the UPLC. Intact peptides were detected in the Orbitrap at a resolution of 70,000. Peptides were selected for MS/MS using an NCE setting of 28, and ion fragments were detected in the Orbitrap at a resolution of 17,500. A data-dependent procedure that alternated between one MS scan followed by 20 MS/MS scans was used for the top 20 precursor ions above a threshold ion count of 2E4 in the MS survey scan with 15.0 s dynamic exclusion. The electrospray voltage applied was 2.0 kV. Automatic gain control (AGC) was used to prevent overfilling of the ion trap. Further, 5E4 ions were accumulated for generation of MS/MS spectra. For each MS scan, the m/z scan range used was 350 to 1,800.

In each LC-MS run, we normalized peptide ratios so that the median of their logarithms was zero, which corrected for unequal protein loading, assuming that the majority of proteins showed no differential regulation. Protein ratios were calculated as the median of all SILAC peptide ratios, minimizing the effect of outliers. We normalized the protein ratios to correct for unequal protein amounts.

### Western blot analysis

Cells were collected, and the core histones from whole cell lysates were extracted.30 μg of proteins was electrophoresed by SDS–PAGE and then transferred to nitrocellulose membranes.The proteins were probed firstwith primary antibodies against H3K23ac, H3K18ac, H4K8ac, and tubulin from PTM BioLab, Inc. (Hangzhou,China) and then with horseradish peroxidase-coupled secondary antibodies at1:5000 dilution(Thermo, Pierce,USA).Proteins were visualized withth eantibodies usingan enhanced chemiluminescence kit from AmershamBiosciences(UK).

### Database search

The resulting MS/MS data was processed using MaxQuant with an integrated Andromeda search engine (v.1.4.1.2). Tandem mass spectra were searched against the SwissProt (20,274 sequences) database concatenated with a reverse decoy database. Trypsin/P was specified as the cleavage enzyme, allowing up to three missed cleavages and five modifications per peptide in addition to five charges. Mass error was set to 10 parts per million (ppm) for precursor ions and 0.02 Da for fragmented ions. Carbamidomethylation on the cysteine residues was specified as a fixed modification, while oxidation on methionine residues, acetylation on lysine residues, and acetylation on the N-terminus of the protein were specified as variable modifications. The false discovery rate (FDR) thresholds for the protein, peptide, and modification sites were set at 1%. Minimum peptide length was set at seven residues. All of the other parameters in MaxQuant were set to their default values. Finally, the site localization probability was set as greater than 0.75.

### Gene Ontology (GO) annotation

According to GO annotation information of identified Kac proteins, we calculated the number of quantifiable proteins or identified Kac proteins in each GO term of level 2. Each of the proteins identified to be differentially expressed in the HCT-8 cells following FK228 treatment was further analyzed in terms of its known functions in the current literature. GO annotation for each protein was derived from the UniProt-GOA database (http://www.ebi.ac.uk/GOA/). To do so, the identified protein ID was converted to its UniProt ID and then mapped to its specific GO IDs. For the proteins not annotated by the UniProt-GOA database, InterProScan soft was used to annotate the protein’s functional GO based on the protein sequence alignment method. The differentially expressed proteins were then classified by their GO annotation based on three categories: biological process, cellular component, and molecular function.

### Kyoto Encyclopedia of Genes and Genomes (KEGG) pathway annotation

KEGG pathway annotation was used to investigate the primary pathways affected by the proteomic changes in the FK228-treated cells. Notably, KEGG pathway analysis mainly focuses on the following pathways: metabolism, genetic information processing, environmental information processing, cellular processes, rat diseases, and drug development. Using the KEGG automatic annotation server (KAAS), we annotated each differentially expressed protein based on its KEGG database description. These results were then mapped using the KEGG online service tool KEGG mapper.

### GO/KEGG pathway functional enrichment analysis

Fisher’s exact tests were used to evaluate the enrichment or depletion (two-tailed test) of specific annotation terms among members of the resulting protein clusters. Any terms with adjusted p-values of less than 0.05 in any of the clusters were treated as significant.

### GO/KEGG pathway functional enrichment

For further hierarchical clustering, we collated all of the categories obtained after enrichment along with their p-values. The categories enriched in at least one of the clusters with a *P* value of less than 0.05 were then identified. This filtered *P* value matrix was then transformed by the following function: X = −log(*P* value), and the X values for each category were z-transformed. These z scores were then clustered using one-way hierarchical clustering (Euclidean distance, average linkage clustering) with Genesis software(Genesis Company, Hyderabad, Indian).

## Conclusions

Using SILAC labeling and affinity enrichment followed by high-resolution LC-MS/MS analysis, we successfully revealed the comprehensive changes in the histone lysine acetylation profile in HCT-8 cells following FK228 treatment. These changes were quantified, revealing that FK228-induced differential lysine acetylation changes in the core histone proteins likely alter the downstream transcription of a wide variety of genes as well as their subsequent protein expression. Bioinformatic analysis further indicated that FK228 may function to regulate protein expression in HCT-8 cells by effecting multiple pathways and protein complexes outside of its role as an HDAC inhibitor. These results significantly deepen our understanding of the underlying mechanism of FK228 in colon cancer HCT-8 cells and provide further information that can be used to determine how best to utilize FK228 in cancer therapy.

## Additional Information

**How to cite this article**: Wang, T.- *et al.* Treating Colon Cancer Cells with FK228 Reveals a Link between Histone Lysine Acetylation and Extensive Changes in the Cellular Proteome. *Sci. Rep.* **5**, 18443; doi: 10.1038/srep18443 (2015).

## Supplementary Material

Supplementary Figure S1

Supplementary Table S1

Supplementary Table S2

Supplementary Table S3

## Figures and Tables

**Table 1:** Summary of Identified Kac Sites and Quantifiable Changes in HCT-8 Cells in Response to FK228 Treatment.

Histone and modified sites	Histone name	Modified sequence	Quantifiable changes of peptide	Report state
P33778_13	Histone H2B type 1-B	_K(ac)GSK(ac)K(ac)AITK_	7.8	Report
P33778_16	Histone H2B type 1-B	_K(ac)GSK(ac)K(ac)AITK_	7.8	Report
P33778_17	Histone H2B type 1-B	_K(ac)GSK(ac)K(ac)AITK_	7.8	Report
Q99877_6	Histone H2B type 1-N	_PEPSK(ac)SAPAPK_	2.8	Report
P06899_121	Histone H2B type F-S	_AVTK(ac)YTSAK_	0.23	
P06899_6	Histone H2B type F-S	_PEPAK(ac)SAPAPK(ac)K(ac)GSK_	2.5	Report
P06899_12	Histone H2B type F-S	_PEPAK(ac)SAPAPK(ac)K(ac)GSK	9.6	Report
P06899_16	Histone H2B type F-S	_K(ac)GSK(ac)K(ac)AVTK_	4.4	Report
P06899_17	Histone H2B type F-S	_K(ac)GSK(ac)K(ac)AVTK_	4.4	Report
P06899_21	Histone H2B type F-S	_GSK(ac)K(ac)AVTK(ac)AQK_	4.9	Report
P62805_9	Histone H4	_GGK(ac)GLGK(ac)GGAK(ac)R_	7.9	Report
P62805_13	Histone H4	_GLGK(ac)GGAK(ac)R_	2.2	Report
P62805_17	Histone H4	_GLGK(ac)GGAK(ac)R_	2.2	Report
P68431_19	Histone H3.1	_K(ac)QLATK(ac)AAR_	5.2	Report
P68431_24	Histone H3.1	_QLATK(ac)AAR_	3.6	Report
Q5QNW6_16	Histone H2B type 2-F	_K(ac)GSK(ac)K(ac)AVTK_	4.4	Report
Q5QNW6_17	Histone H2B type 2-F	_K(ac)GSK(ac)K(ac)AVTK_	3.3	Report
Q5QNW6_21	Histone H2B type 2-F	_K(ac)AVTK(ac)VQK_	3.3	Report
Q5QNW6_6	Histone H2B type 2-F	_PDPAK(ac)SAPAPK(ac)K_	6.7	Report
Q99879_17	Histone H2B type 1-M	_K(ac)GSK(ac)K(ac)AINK_	3.8	Report
Q99879_21	Histone H2B type 1-M	_K(ac)AINK(ac)AQK(ac)K_	3.8	Report
Q99879_24	Histone H2B type 1-M	_K(ac)AINK(ac)AQK(ac)K_	6.1	Report
Q99879_13	Histone H2B type 1-M	_K(ac)GSK(ac)K(ac)AINK_	5.1	Report
Q99879_16	Histone H2B type 1-M	_K(ac)GSK(ac)K(ac)AINK_	5.1	Report
Q99880_16	Histone H2B type 1-L	_K(ac)GSK(ac)K(ac)AVTK_	4.4	Report
Q99880_13	Histone H2B type 1-L	_PELAK(ac)SAPAPK(ac)K(ac)GSK_	4.4	Report
Q99880_6	Histone H2B type 1-L	_PELAK(ac)SAPAPK(ac)K(ac)GSK_	4.9	Report
Q99880_12	Histone H2B type 1-L	_PELAK(ac)SAPAPK(ac)K(ac)GSK_	6.3	Report
