# Supplementary figures and images for: Treating Colon Cancer Cells with FK228 Reveals a Link between Histone Lysine Acetylation and Extensive Changes in the Cellular Proteome
Source: Sci Rep. 2015 Dec 17;5:18443. doi: 10.1038/srep18443 (PMC4682073; doi:10.1038/srep18443)

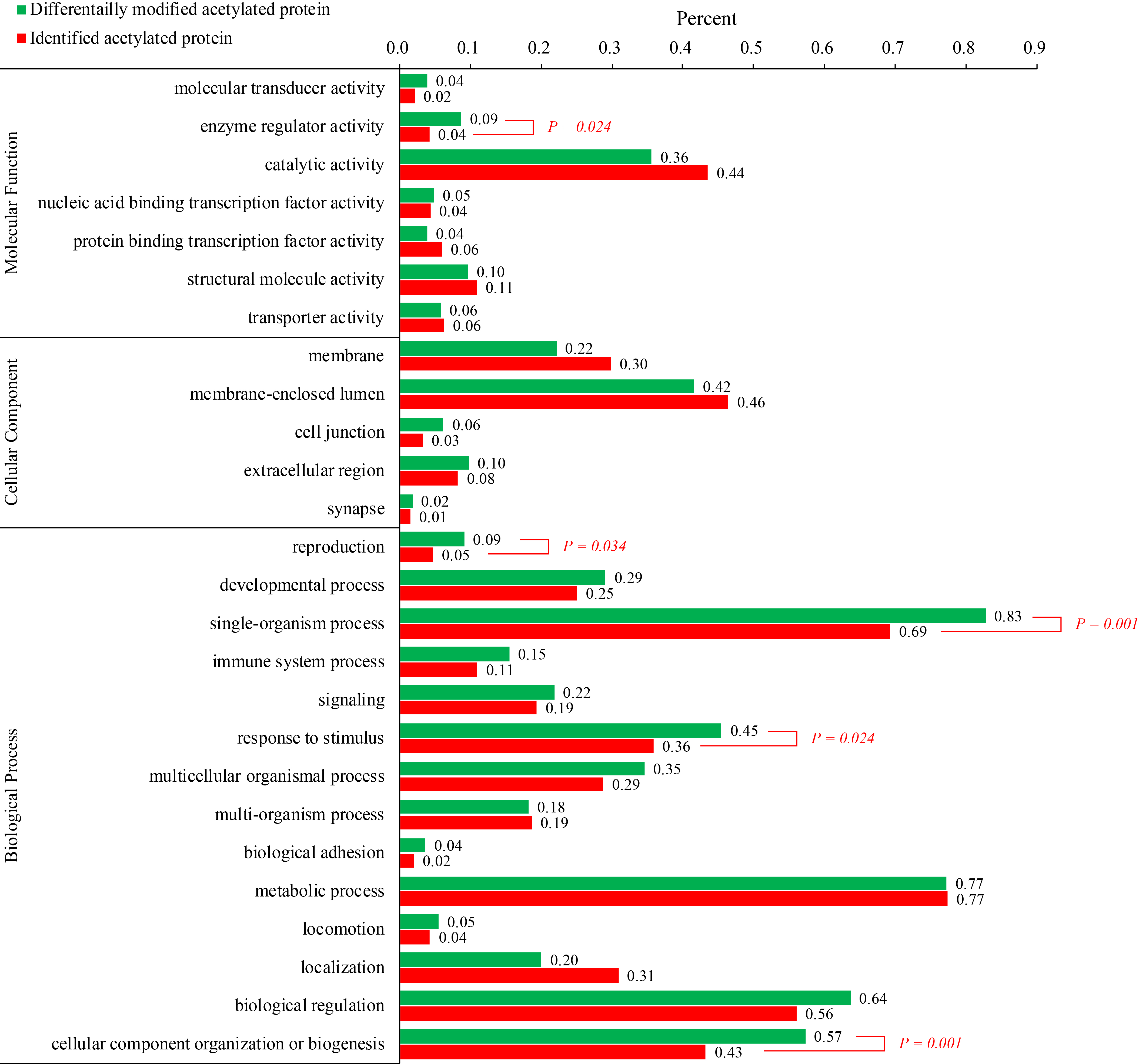

Supplement: Supplementary Figure S1 [file srep18443-s1.jpg]
